# Clinical Reflections

**Published:** 1881-12

**Authors:** Ad. Lippe

**Affiliations:** Philadelphia


### CLINICAL REFLECTIONS.

AD. LIPPE, M. D., PHILA.

CASE 1. On the 28th of August, 1881, I was called to see a gentleman, age 50 years, who had just come to town for treatment. He had been at Bedford Springs on the Alleghany mountains, to escape the summer heat. Not in robust health and having suffered nine years ago from a severe attack of pneumonia (right side) for which we had then treated him successfully, he now was aware of having been unprotected against a sudden change of temperature on the 24th of August while driving in the country. That evening he had a severe chill, was so uncomfortable in a horizontal position, suffering severe dyspnœa, that he was compelled to sit up in a chair all night. Thinking that it might be an attack of asthma, which is hereditary in his family, and that the attack would pass off in a day or two, he remained in his room till the 27th, when by advice of a medical friend, who did not proscribe for him, he was taken home. I found him 10 A. M. sitting on a sofa, unable to lie down; respiration 36, pulse 120 in a minute; asked him to rise that I might examine his chest, he did so and attempted to make a few steps, this slight exertion caused very rapid breathing and cough, and for some minutes he was unable to talk. The examination revealed the existence of severe pneumonia, the left lower lobe was hepatized and the inflammation had progressed upwards. The pulse was small and hard ; no appetite whatever ; mouth dry, tongue furred whitish yellow ; much thirst but drinking and talking caused more cough, the soreness in the chest was increased by the slightest movement, and the dyspnœa by lying down ; there was no expectoration ; the urine which had been very profuse, while he was drinking daily a very small amount of Bedford water, was now very scanty, very dark, no sediment ; bowels had not been moved for some days ; skin hot and dry ; aggravation of fever and nervous restlessness between the hours of 9 P. M. and 1 A. M. He received one dose of *Phosphorus* CM (F.) at 10 A. M. When visited in the evening he was found slightly better ; was able to lie down for a short time towards evening, and reported himself feeling better, his breathing was less labored and not quite so frequent. This improvement continued for six days, when he was able to sleep in bed, and to take some food : on the seventh day the improvement discontinued and he received another dose of *Phosphorus*, this time M (F.). The improvement was now more rapid. On the 9th day he slept quietly all night, the perspiration which had been very profuse diminished, his appetite was good; on the 12th day he drove out, when he now coughed, he expectorated white, salty-tasting mucus, and was able to go to Maryland on the 18th day. He now enjoys his usual health.

*Comments.* This case shows, like many others, what results can be obtained if we only follow the strict tenets of our school, and the more grave the case is the better are the results of strictly homœopathic treatment. From the symptoms above stated there could be no other similar remedy found than *Phosphorus* ; as to the dose, that is solely a matter of experience; a lower potency would probably have also cured this grave case, but, as far as our experience goes, not quite as quickly and surely. Of late years the attempt has been made to prove, *a priori*, that such doses as cured this case have no curative powers; one doubter sees no medicine beyond the 6th potency, another observer can not imagine that any medicinal virtue exists beyond the 3d potency and still others doubt anything they can not see, taste or smell. We now call the attention of these doubters to an opinion offered the profession by the late Carroll Dunham, we appeal to his address before the " World's Homœopathic Convention of 1876," where he says :

" *Our opponents claim to have demonstrated again and again that there is nothing in our potentized preparations. The reasoning of Thomson touching the size of molecules furnishes them with a welcome argument against the possibility of any drug potency existing in even our medium attenuations. And these arguments have strongly inflvenced many of our own school whose personal experience and observa-*

*tion had not compelled opposite convictions. But let me say that proofs of a negative kind in any matter which can be determined only by experiment are fallacious, and a dangerous dependence. I do not despair of seeing before many years, from some old-school authority or some non-medical investigator, a demonstration of the medicinal power of homœopathic potencies; and I warn such of my colleagues as have been influenced by the arguments of our opponents, against the chagrin they will feel when they shall be outflanked on this point; when to unbelieving homœopathists shall be presented, by experimenting allopaths, a demonstration of the drug-power inherent in homœopathic attenuations."*

The prophetic words then uttered have been fulfilled fully and to every *scientific* man's satisfaction. We call the attention of such colleagues, as were warned by our late C. Dunham, to Professor Jæger's Neural Analysis, and to Professor Pasteur's vaccine discoveries. It becomes obvious that Hahnemann's dynamization doctrine has been fully proven to be correct by Profs. Jæger and Pasteur, and can not possibly be represented as a theory or as a supplementary principle, * and we may say here that the declaration of Principles accepted by the International Hahnemannian Association does not differ in the least from Hahnemann's teachings as we find them in his "Organon of the Healing-Art."

No matter how many "*brainy* men"† constitute themselves as judges and they forming a court of final appeal, a tribunal which summons Hahnemann and his Organon to appear before them to be by them judged; no matter by what sophistry these "*brainy* men" attempt to show that Hahnemann's dynamization doctrine does not constitute an essential and necessary part of homœopathy, but merely amounts to but a supplementary principle; this tribunal will find that they have no jurisdiction over the case—that both the experience of Hahnemann's true followers and the experiment of Old-School physicians, have long ago entered judgment in favor of the great Philosopher, who arrived at his dynamization doctrine by his strict inductive method, and is now endorsed by modern scientists, these "*brainy* men" may as well learn that there can be no appeal from the judgment entered long before they attempted to try his case.

CASE 2. A lady 35 years old, of good robust constitution, presented herself, showing the palm of the right hand. The skin had become dry, vesicles formed with intolerable burning and itching; on rubbing them they emitted a yellow lymph; similar vesicles began to form on the fingers, the left hand began to itch in the palm of it,

---

***Hahnemannian Monthly*, October, 1881, Page 627. †Ibid, p. 626.

much worse at night depriving her of sleep; had applied various fatty substances but they made it worse, and now at last a scab, formed in the palm of the right hand after the vesicles had broken. Otherwise she was in perfect health. There was no remedy more similar then *Ranunculus bulbosus.* She therefore received one dose of *Ran. bulb.* 65M (F.). The second night after this single dose had been taken the burning and itching ceased, every vesicle dried up, and in three weeks the remaining dryness of the palms of the hands had also disappeared.

*Comments.* There were no supplementary or external means used—the similar remedy alone, without any external applications, proved itself sufficient to cure the case and that in a single dose.

CASE 3. A lady, past 80 years of age, subject to attacks of asthma, after a preliminary attack of Rose (Hay) fever, was taken sick while near West Point. Came to New York, and was not relieved of the nightly asthma and cough by her usually quick acting remedy—*Lachesis.* Dr. C. Lippe, of New York, waited on her and as *Lachesis* evidently did not relieve her, and as he had tested the excellent indications for the use of *Aralia racemosa,* given by Dr. Burnett, of London, and as these very symptoms were present.

Oppressed breathing all day, as if a weight was lying on the chest; the dyspnœa became much worse on lying down at night; after falling asleep in a sitting position, she awakened with a violent cough, and was only relieved after she had finally raised some extremely tenacious and frothy mucus. *Aralia* 30 was administered, dissolved in water, by spoonfuls, once in 2 hours. The relief came the first night when all her symptoms were much less severe, as the remedy was not then suspended, but contrary to orders had been continued all day, the second night was again worse, no medicine was ordered and she improved so much that she could return to Philadelphia, her home, in 4 days. The improvement of this one dose of *Aralia* continued for 6 days, there was more and freer expectoration, but as the sputa became much moie tenacious and the dyspnœa correspondingly more severe, just as before, worse when lying down and cough worse when awakening from sleep, I now gave her one single dose of *Aralia* CM (Swan). As before, after taking *Aralia* 30, the dyspnœa improved very much and she slept much longer before the cough awakened her. This was the last dose of *Aralia* necessary to relieve all these characteristic symptoms in about 10 days.

*Comments.* Cough when awakening from sleep is strongly under *Lachesis, Kali bichr., Sulph., Nitr. ac., etc.,* but *Aralia* is more similar to *Lachesis,* as both have dyspnœa when lying down and cough on awakening; *Lachesis* has not the tenacious frothy mucus of *Aralia; Kali bichr.* has relief of cough when lying down, but the tenacious mucus differs in both remedies, *Kali bichr.* has tenacity, the mucus is expectorated and cannot be easily detached, the mucus is stringy; the *Aralia* mucus is tenacious and frothy but after it has been detached from the bronchia with great difficulty, after protracted coughing it is expectorated easily. *Sulphur* has a peculiar cough after a long sleep, under *Nitr. ac.,* the cough awakens him at 4 A. M. regularly.

Again, this case shows plainly the correctness of Hahnemann's doctrine of dynamization, as accepted by the I. H. A. We have a public declaration of the General Editor of the *Hahnemannian Monthly,* a journal published by the Hahnemann Club, October, 1881, last line of page 628 and page 629 where he says, "We CAN *practice a pure Homœopathy without this supplementary principle,* (*Hahnemann's dynamization theory*), *but we don't want to be compelled to do it.*" We can't!

